# It Takes a Village: Discovering and Isolating the Nitrifiers

**DOI:** 10.3389/fmicb.2020.01900

**Published:** 2020-08-11

**Authors:** Christopher J. Sedlacek

**Affiliations:** Division of Microbial Ecology, Centre for Microbiology and Environmental Systems Science, University of Vienna, Vienna, Austria

**Keywords:** nitrification, nitrogen cycle, ammonia oxidizers, nitrite oxidizers, comammox

## Abstract

It has been almost 150 years since Jean-Jacques Schloesing and Achille Müntz discovered that the process of nitrification, the oxidation of ammonium to nitrate, is a biological process carried out by microorganisms. In the following 15 years, numerous researchers independently contributed paradigm shifting discoveries that formed the foundation of nitrification and nitrification-related research. One of them was Sergei Winogradsky, whose major accomplishments include the discovery of both lithotrophy (in sulfur-oxidizing bacteria) and chemoautotrophy (in nitrifying bacteria). However, Winogradsky often receives most of the credit for many other foundational nitrification discoveries made by his contemporaries. This accumulation of credit over time is at least in part due to the increased attention, Winogradsky receives in the scientific literature and textbooks as a “founder of microbiology” and “the founder of microbial ecology.” Here, some light is shed on several other researchers who are often overlooked, but whose work was instrumental to the emerging field of nitrification and to the work of Winogradsky himself. Specifically, the discovery of the biological process of nitrification by Schloesing and Müntz, the isolation of the first nitrifier by Grace and Percy Frankland, and the observation that nitrification is carried out by two distinct groups of microorganisms by Robert Warington are highlighted. Finally, the more recent discoveries of the chemolithoautotrophic ammonia-oxidizing archaea and complete ammonia oxidizers are put into this historical context.

## Introduction

It has been almost 150 years since the discovery of microbially mediated nitrification (the oxidation of ammonium to nitrate), and ever since scientists have been working toward identifying and characterizing the microorganisms responsible. The ability to regulate the process of nitrification in the environment is essential in a world battling to reduce greenhouse gas emissions, prevent the eutrophication of aquatic ecosystems, and increase the efficiency of drinking and waste water treatment (Galloway et al., 2003, 2017; Fields, 2004; Erisman et al., 2008; Houlton et al., 2019). With that in mind, the study of nitrification and the microbes that carry this process out is arguably more relevant than ever. Here, a look back at several groundbreaking discoveries that form the foundation for nitrification-related research is presented.

Many independent researchers and research groups contributed to these groundbreaking discoveries, but one researcher in particular receives the lion’s share of the credit for the initial work surrounding nitrification and nitrifying microorganisms, the Russian microbiologist Sergei Winogradsky. Winogradsky is often broadly credited with the discovery of nitrification, the isolation of the first nitrifiers, and the observation that nitrification occurs as a two-step process. Interestingly, he is most likely not responsible for any of these particular discoveries (albeit determining who really had the first pure nitrifier culture is not without controversy). This accumulation of credit is in part a consequence of the increased attention Winogradsky receives in the scientific literature and textbooks – due to his impressive body of work. In fact, Winogradsky’s seminal papers on nitrification cite the foundational work performed by the lesser known researchers highlighted here (Winogradsky, 1890, 1891).

Winogradsky did of course make numerous pioneering contributions to the field of nitrification and to the microbiological sciences as a whole. While at the Swiss Polytechnic Institute in Zurich, he discovered that nitrifiers were chemoautotrophic (first example of microbial autotrophy), proposed that nitrification was performed by a particular type of microorganism, championed the enrichment culturing technique that is still widely utilized today and possibly isolated the first nitrite-oxidizing bacterium (Winogradsky, 1890). Combined with his discovery of microbial lithotrophy while working with the sulfur-oxidizing *Beggiota* at the University of Strasbourg (Winogradsky, 1887; Doetsch, 1960), and his doctrine of pleomorphism (Winogradsky, 1936; Doolittle, 2013), it is easy to see why he is referred to as a “founder of microbiology” and “the founder of microbial ecology.” Consequently, his life and scientific discoveries have been well documented several times over (Waksman, 1953; Penn and Dworkin, 1976; Zavarzin, 2006; Ackert, 2007; Dworkin, 2011). So, while the works of Winogradsky will be mentioned here for context, his (and his longtime research assistant, Viselli Omelianski’s) work will not be the focus of this piece.

Instead, some light will be shed on a few of the other less well known but nonetheless critical researchers responsible for key nitrification-related discoveries between 1877 and 1892, which often get credited to, or grouped with, Winogradsky’s accomplishments. Their discoveries are put into a timeline highlighting some of the large scientific leaps made in nitrification research during this early period. In addition, this timeline was extended to include the more recent paradigm shifting discoveries of the ammonia-oxidizing archaea and complete ammonia oxidizers.

## Nitrification Is a Biotic Process

Up until the late 1870s, ammonium and nitrates held quite of bit of mystery in both agricultural and drinking water quality research. For instance, it was unknown how nitrates were replenished in unfertilized soils. Common hypotheses centered around abiotic chemical reactions, such as reactions of atmospheric nitrogen and oxygen, organic nitrogen with oxygen or ozone, or ammonia with ferric oxide (reviewed by Warington, 1878a). In addition, it was Edmund Davy, an Irish medical doctor and professor of forensic medicine at the Royal College of Surgeons in Ireland, who first observed that ammonium concentrations decreased while nitrite (and he assumed nitrate) increased over time in sewage contaminated drinking water. Therefore, in order to rule out sewage contamination, Davy argued that drinking water should be tested for not only just ammonium but also nitrite and nitrate (Davy, 1883). Notably, although Davy did not attempt to answer whether nitrification was a biotic process or not, he detailed how the process of nitrification: (1) is inhibited by high amounts of organic matter, (2) requires air or free oxygen, and (3) proceeds at an optimal temperature of between 21 and 27°C.

The first person to hypothesize that ammonia oxidation may in fact be a biological process was the French chemist/microbiologist Luis Pasteur, who in 1862 suggested that ammonia may be oxidized to nitrate in a similar manner as alcohol is oxidized to acetic acid (Pasteur, 1862). A decade later, Alexander Müller, a German agricultural chemist, would put forward that ammonia oxidation must be performed by microorganisms. While investigating water quality from wells in Berlin, Müller noted that ammonium was stable in sterilized solutions in the laboratory but readily nitrified in natural waters (Müller, 1875). Even with these observations, it would be ~25 years (1877) after Pasteur’s initial hypothesis until two French agricultural chemists working in Paris, Jean-Jacques Schloesing and Achille Müntz, demonstrated that the oxidation of ammonium in sewage and soils was indeed a microbially mediated process (Schloesing and Müntz, 1877a,b).

In order to demonstrate the biological nature of nitrification, Schloesing and Müntz slowly passaged liquid sewage through an artificial soil matrix column consisting of sterilized sand and powered chalk. With this experimental setup, they were able to make four key observations: (1) there was an initial lag phase, (2) as the ammonia concentration decreased the nitrate concentration increased in the filtrate, (3) nitrification irreversibly ceased when the column was exposed to chloroform or high heat, and (4) nitrification could be restarted by adding small amounts of soil washings (Schloesing and Müntz, 1877a,b). These results were independently confirmed by the English agricultural chemist Robert Warington who was investigating the nitrification ability of garden soil at the (still operational today) Rothamsted experimental station in Harpenden England. Warington was also able to demonstrate that soil added to dilute solutions of ammonium would produce nitrate, and that these nitrifying solutions could seed new nitrifying solutions – with this, nitrifier enrichment/cultivation was born (Warington, 1878b). Together, these seminal studies kick-started the study of microbial nitrification.

Interestingly, one of the still remaining open questions in nitrification research became a topic of debate during these early years. Several researchers published observations very early on as to whether or not direct sunlight inhibited the process of nitrification, with varied results (Warington, 1878b, 1879, 1884; Davy, 1883; Munro, 1886). However, the effect of 24-hour illumination on nitrification could not be studied, as the incandescent light bulb was still being developed.

## Nitrification Is a Two-Step Process

In 1879, Warington made the first observation that nitrification proceeds as a two-step process, involving the oxidation of ammonia to nitrite and the oxidation of nitrite to nitrate. Here, Warington described and propagated individual ammonia‐ and nitrite-oxidizing enrichment cultures. In addition, he observed that fully nitrifying cultures only built up nitrite as an intermediate if the rate of ammonia oxidation was sufficiently high (Warington, 1879). The two-step process of nitrification would be later confirmed by the English chemist John Munro working at the College of Agriculture at Downton (Munro, 1886). These observations were made well before the isolation of the first nitrifier in 1890. However, the hypothesis at the time was that the ammonia‐ and nitrite-oxidizing cultures represented different life phases or character traits of the single nitrifying microorganism (Warington, 1884, 1891).

## Nitrifier Isolation Attempts

Once it was confirmed that nitrification was a microbially mediated process, the race was on to be the first researcher to isolate and characterize the microorganism(s) responsible. Over the course of the next ~15 years (1877-1890), several researchers made claims that they isolated cultures that had (or previously had) full nitrifying capabilities (the ability to oxidize ammonium to nitrate). At the time, it was still hotly debated whether microorganisms were pleomorphic – able to radically change their abilities or characteristics under different circumstances, sometimes irreversibly in short periods of time (Winogradsky, 1936; Doolittle, 2013). With the pleomorphic hypothesis in mind, some researchers observed that nitrifying microorganisms irreversibly lost their nitrifying capabilities through the process of isolation. These studies attempted to isolate single nitrifier colonies on solid growth medium containing high amounts of organic carbon. In a somewhat ironic twist, this exact approach (growth in or on medium containing high amounts of organic carbon) is now common practice in order to detect heterotrophic contaminants in pure nitrifier cultures. However, because each researcher isolated their own unique environmental cultures and some of these cultures had seemingly lost their nitrifying capabilities by the time of publication, independent conformational studies were difficult if not impossible.

As a notable exception, the German chemist Wilhelm Heraeus, working at the Institute of Hygiene in Berlin, published in 1886 that in addition to cultures, he freshly isolated from sites around Hanau Germany, several well-known bacterial cultures, including *Bacillus anthracis* and *Bacillus ramosus* had nitrifying capabilities. In his publication, Heraeus notes that some of these isolated cultures produced detectable but not quantifiable amounts of nitrite and nitrate when cultured in dilute urine (Heraeus, 1886). As these *Bacillus* strains were more widely available to the scientific community, others could attempt to replicate the results. Indeed, the English agricultural chemist Percy Frankland attempted but could not independently verify Heraeus’s results. Frankland determined that Heraeus was most likely detecting small amounts of nitrate present in the urine, and that the *Bacillus* cultures were reducing this nitrate to nitrite. By inoculating the *Bacillus* cultures into ammonium solutions instead of dilute urine, Frankland was able to refute Heraeus’s claims as no nitrite or nitrate was produced (Frankland, 1888). It would not be until 1890 that Percy and Grace Frankland, two English scientists working in Dundee Scotland would publish about the isolation of what is most likely the first pure nitrifier (ammonia-oxidizing) culture (Frankland and Frankland, 1890). This breakthrough was made possible by the combined expertise of Grace as a bacteriologist and Percy as an agricultural chemist.

## Isolation of the First Nitrifiers

For years researchers (including the Frankland’s) failed to isolate actively nitrifying cultures using Robert Koch’s solid medium isolation techniques (Koch, 1882). However, Warington, Winogradsky, and the Frankland’s were all able to establish and propogate nitrifying enrichment cultures (Warington, 1878b; Frankland and Frankland, 1890; Winogradsky, 1890). Ultimately Frankland and Frankland were the first to isolate a pure nitrifier (ammonia-oxidizing bacteria) from their enrichment cultures. In order to achieve a pure culture, they employed a system of serial dilutions with very low inoculum over the period of several years (Frankland and Frankland, 1890). This method of serial dilution to extinction is a tried and true method that is still used for the (painstakingly slow) isolation of nitrifiers today.

In 1890, both Winogradsky as well as Frankland and Frankland would both claim that they were the first to have a pure nitrifier culture (Frankland and Frankland, 1890; Winogradsky, 1890). However, at this time, Winogradsky’s pure culture was still a fully nitrifying culture, and he would later disclose through personal communications (~40 years later) that his nitrifying cultures were suitable for nitrification studies but not pure in the strictest sense of the word (Hanks and Weintraub, 1936). With the more recent discovery of complete ammonia oxidizers in mind, it is tempting to speculate that Winogradsky did in fact have a pure culture of a complete ammonia oxidizer capable of fully nitrifying. However, based on his work in the years to come, this culture was much more likely a co-culture of ammonia‐ and nitrite-oxidizing bacteria. Winogradsky was able to separate the ammonia‐ and nitrite-oxidizing bacteria just a year later (Winogradsky, 1891).

In contrast to other researchers before them, both the team of Frankland and Frankland as well as Winogradsky took many steps in order to ensure they had an actively nitrifying pure culture. Both noted that pure nitrifying cultures did not produce any growth on solid media, and that the nitrifying liquid medium remained clear throughout the entirety of nitrification. Additionally, they monitored and quantified the conversion of ammonium into nitrite and nitrate (Frankland and Frankland, 1890; Winogradsky, 1890, 1891).

Although Frankland and Frankland were confident that they had isolated the microorganism responsible for nitrification, the isolate only oxidized ammonium to nitrite when in its pure form (Frankland and Frankland, 1890). Warington had observed a similar phenomenon years ago, noting that after several passages in the laboratory nitrifying cultures would often produce nitrite rather than fully nitrifying (Warington, 1879, 1884). There were several hypotheses to explain this partial nitrification: (1) the oxidation of nitrite to nitrate was completed by a second uncultured microorganism, (2) the conditions for full ammonia oxidation were not met in the laboratory, or (3) part of the microorganism’s physiology present when in soil was lost rapidly during laboratory cultivation. In hindsight, Warington as well as Frankland and Frankland, were unknowingly separating ammonia‐ and nitrite-oxidizing bacteria through the use of different growth mediums and transfer techniques.

As is the case with the ammonia-oxidizing bacteria, determining which researcher(s) truly isolated the first nitrite-oxidizing bacteria, is not without uncertainty. Again, there were researchers that claimed to isolate nitrite-oxidizing bacteria that quickly lost their nitrite oxidation abilities once grown and isolated on or in organic growth medium (Beijerinck, 1914), which were most likely not pure nitrite-oxidizing bacteria. In addition, some seemingly pure cultures were later discovered to be highly enriched cultures with only a few bacterial members (Burri and Stutzer, 1895). But there are about 10 research groups, who between 1900 and 1960 claim to have purified nitrite-oxidizing bacteria, which did not produce noticeable growth when inoculated on or in growth medium containing high amounts of organic matter (reviewed by Zavarzin and Legunkova, 1959; but missing Fred and Davenport, 1921). As with the ammonia oxidizers, Winogradsky was the first to claim a pure nitrite-oxidizing culture (Winogradsky, 1891). Unfortunately, unlike with the isolation of the first ammonia oxidizers, where there was a clear difference between theoretically pure cultures that produce nitrite versus those that produce nitrate from ammonium; here, all theoretically pure nitrite oxidizer cultures share the same reported physiology (nitrite consumption and nitrate production). Therefore, without preserved cultures, it is difficult if not impossible to say with certainty which cultures contained no heterotrophic contaminants.

Nitrite oxidizer isolation represents another close call for Robert Warington. After being the first to observe the two steps of nitrification (even if the significance of the observation was not immediately clear), he spent years attempting to isolate nitrite-oxidizing microorganisms through the serial dilution technique shown to be successful by Frankland and Frankland with ammonia oxidizers, but to no avail. He would end his scientific career before he was able to produce a pure nitrite-oxidizing isolate (Warington, 1891).

## New Nitrifiers Discovered In The 21^st^ Century

Since the inaugural era of nitrification research discussed so far, there has been a wealth of studies that have propelled the field of nitrification forward during the subsequent ~100 years. These advances include (but are not limited to) the (1) discovery of many phylogenetically and physiologically distinct ammonia‐ and nitrite-oxidizing bacteria, (2) a deeper understanding of nitrifier (eco)physiology/enzymology, and (3) extensive environmental surveys. These discoveries, isolations, and characterizations have previously been thoroughly reviewed throughout the years (Watson et al., 1981, 1989; Prosser, 1989; Koops and Pommerening-Röser, 2001; Kowalchuk and Stephen, 2001; Koops et al., 2006; Arp et al., 2007; Daims et al., 2011; Stahl and de la Torré, 2012).

Even with this immense increase in the diversity of known nitrifiers, up until just over 15 years ago, it was widely accepted that (1) there was a complete division of labor in nitrification and (2) nitrification was carried out solely by bacteria. Then, over the course of a 10-year period both of these century-old paradigms were shattered with the discovery and isolation of ammonia-oxidizing archaea and complete ammonia oxidizers. Just as with the discovery and isolation of the ammonia‐ and nitrite-oxidizing bacteria, these discoveries too, took a village.

### Ammonia-Oxidizing Archaea

In 2004, two independent research groups identified putative ammonia monooxygenase genes (the main functional genes used to identify ammonia oxidizers) in genomic sequence material belonging to a *Crenarchaeota* microorganism (Treusch et al., 2004; Venter et al., 2004).

Almost immediately, these findings were followed key genomic comparisons (Schleper et al., 2005) and environmental survey studies (Francis et al., 2005). Amazingly, the first isolated ammonia-oxidizing archaea culture, *Nitrosopumilus maritimus*, was published within the next year (Könneke et al., 2005), and the first genome of an ammonia-oxidizing archaea (*Candidatus Cenarchaeum symbiosum*) was published soon after (Hallam et al., 2006a,b). Since these works, there have been hundreds of studies focusing on the diversity, physiology, and environmental distribution of ammonia-oxidizing archaea, highlighting to what extent they have changed the field of nitrification in such a short time (reviewed by Erguder et al., 2009; Schleper and Nicol, 2010; Urakawa et al., 2011; Hatzenpichler, 2012; Prosser and Nicol, 2012; Stahl and de la Torré, 2012; Alves et al., 2018).

### Complete Ammonia Oxidizers

Although a complete ammonia oxidizer had never been described, their existence was by no means a new hypothesis. The whole field of nitrification began with the belief that nitrification was completed by one microorganism (a nitrifying ferment). In addition, a recent theoretical kinetic study hypothesized that complete ammonia oxidizers would be found in environments that select for slow growing but high yield microorganisms (Costa et al., 2006). In 2015, just 10 years after the discovery of the ammonia-oxidizing archaea, the existence of complete ammonia oxidizers was published in parallel by two research groups. Both studies yielded metagenomic evidence for, and enrichment cultures of, a complete ammonia oxidizer (Daims et al., 2015; van Kessel et al., 2015). Within a year, two additional research groups published evidence supporting these findings (Pinto et al., 2015; Palomo et al., 2016). The isolation of *Nitrospira inopinata*, the first complete ammonia oxidizer brought into pure culture, followed shortly thereafter (Daims et al., 2015; Kits et al., 2017). In the same manner as the discovery of the ammonia-oxidizing archaea before them, studies focusing on the diversity, physiology, and environmental distribution of complete ammonia oxidizers are now increasing at a rapid pace (reviewed by Daims et al., 2016; Palomo et al., 2018; Xia et al., 2018; Koch et al., 2019). It took about 150 years, but the field of nitrification has now come full circle – a fully nitrifying microorganism with the ability to oxidize ammonia all the way to nitrate.

## In Summary

While Winogradsky has certainly earned his title of “the founder of microbial ecology,” the breakthroughs illustrated here highlight the work of many of his often-overlooked contemporaries. Schloeing, Muntz, Warington, Frankland, and Frankland among others, all contributed essential observations and discoveries that now form the foundation of nitrification-related research. With the surprising discoveries of new types of nitrifiers within just the past 15 years, the future of nitrification research looks bright. It will be exciting to see whether there are even more fundamentally different types of nitrifiers including nitrite-oxidizing or complete ammonia-oxidizing archaea out there waiting to be discovered and isolated.

## Author Contributions

The author confirms being the sole contributor of this work and has approved it for publication.

### Conflict of Interest

The author declares that the research was conducted in the absence of any commercial or financial relationships that could be construed as a potential conflict of interest.

## References

[ref1] Ackert L. T. (2007). The “cycle of life” in ecology: Sergei Vinogradskii’s soil microbiology, 1885–1940. J. Hist. Biol. 40, 109–145. 10.1007/s10739-006-9104-6

[ref2] Alves R. J. Minh B. Q. Urich T. Haeseler A. Schleper C. (2018). Unifying the global phylogeny and environmental distribution of ammonia-oxidising archaea based on amoA genes. Nat. Commun. 9:1517. 10.1038/s41467-018-03861-1, PMID: 29666365 PMC5904100

[ref3] Arp D. J. Chain P. S. G. Klotz M. G. (2007). The impact of genome analyses on our understanding of ammonia-oxidizing bacteria. Annu. Rev. Microbiol. 61, 503–528. 10.1146/annurev.micro.61.080706.093449, PMID: 17506671

[ref4] Beijerinck W. (1914). Ueber das Nitratferment und ueber physiologische Artbildung. Folio Microbiol. 3, 91–113.

[ref5] Burri R. Stutzer A. (1895). Ueber einen auf Nährgelatine gedeihenden nitratbildenden *Bacillus*. Central. Bakt. 2, 721–740.

[ref6] Costa E. Pérez J. Kreft J. U. (2006). Why is metabolic labour divided in nitrification? Trends Microbiol. 14, 213–219. 10.1016/j.tim.2006.03.006, PMID: 16621570

[ref7] Daims H. Lebedeva E. V. Pjevac P. Han P. Herbold C. Albertsen M.. (2015). Complete nitrification by *Nitrospira* bacteria. Nature 528, 504–509. 10.1038/nature16461, PMID: 26610024 PMC5152751

[ref8] Daims H. Lücker S. Le Paslier D. Wagner M. (2011). “Diversity, environmental genomics, and ecophysiology of nitrite-oxidizing bacteria” in Nitrification. eds. Ward B. Arp D. Klotz M. (Washington, DC: ASM Press), 295–322.

[ref9] Daims H. Lücker S. Wagner M. (2016). A new perspective on microbes formerly known as nitrite-oxidizing bacteria. Trends Microbiol. 24, 699–712. 10.1016/j.tim.2016.05.004, PMID: 27283264 PMC6884419

[ref10] Davy E. W. (1883). Notes of some observations on nitrification. Proc. R. Ir. Acad. Sci. 3, 242–247.

[ref11] Doetsch R. M. (1960). Historical contribution from 1776 to 1908 by Spallanzani, Schwann, Pasteur, Cohn, Tyndall, Koch, Lister, Schloesing, Burrill, Ehrlich, Winogradsky, Warrington, Beijerinck, Smith, Orla-Jensen. New Brunswick, NJ: Rutgers University Press.

[ref12] Doolittle W. F. (2013). Microbial neopleomorphism. Biol. Philos. 28, 351–378. 10.1007/s10539-012-9358-7

[ref13] Dworkin M. (2011). Sergei Winogradsky: a founder of modern microbiology and the first microbial ecologist. FEMS Microbiol. Rev. 36, 364–379. 10.1111/j.1574-6976.2011.00299.x, PMID: 22092289

[ref14] Erguder T. H. Boon N. Wittebolle L. Marzorati M. Verstraete W. (2009). Environmental factors shaping the ecological niches of ammonia-oxidizing archaea. FEMS Microbiol. Rev. 33, 855–869. 10.1111/j.1574-6976.2009.00179.x, PMID: 19453522

[ref15] Erisman J. Sutton M. Galloway J. Klimont Z. Winiwarter W. (2008). How a century of ammonia synthesis changed the world. Nat. Geosci. 1, 636–639. 10.1038/ngeo325

[ref16] Fields S. (2004). Global nitrogen: cycling out of control. Environ. Health Perspect. 112, A556–A563. 10.1289/ehp.112-a556, PMID: 15238298 PMC1247398

[ref17] Francis C. A. Roberts K. J. Beman J. M. Santoro A. E. Oakley B. B. (2005). Ubiquity and diversity of ammonia-oxidizing archaea in water columns and sediments of the ocean. Proc. Natl. Acad. Sci. U. S. A. 102, 14683–14688. 10.1073/pnas.0506625102, PMID: 16186488 PMC1253578

[ref18] Frankland P. F. (1888). The action of some specific microorganisms on nitric acid. J. Chem. Soc. Trans. 53, 373–391. 10.1039/CT8885300373

[ref19] Frankland P. F. Frankland G. C. (1890). The nitrifying process and its specific ferment. Part I. Philos. Trans. R. Soc. Lond. B 181, 107–128. 10.1098/rstb.1890.0005

[ref20] Fred E. B. Davenport A. (1921). The effect of organic nitrogenous compounds on the nitrate-forming organism. Soil Sci. 11, 389–407. 10.1097/00010694-192105000-00006

[ref21] Galloway J. N. Aber J. D. Erisman J. W. Seitzinger S. P. Howarth R. W. Cowling E. B. (2003). The nitrogen cascade. Bioscience 53, 341–356. 10.1641/0006-3568(2003)053[0341:TNC]2.0.CO;2

[ref22] Galloway J. N. Leach A. M. Erisman J. W. Bleeker A. (2017). Nitrogen: the historical progression from ignorance to knowledge, with a view to future solutions. Soil Res. 55, 417–424. 10.1071/SR16334

[ref23] Hallam S. J. Konstantinidis K. T. Putnam N. Schleper C. Watanabe Y. Sugahara J.. (2006a). Genomic analysis of the uncultivated marine crenarchaeote *Cenarchaeum symbiosum*. Proc. Natl. Acad. Sci. U. S. A. 103, 18296–18301. 10.1073/pnas.0608549103, PMID: 17114289 PMC1643844

[ref24] Hallam S. J. Mincer T. J. Schleper C. Preston C. M. Roberts K. Richardson P. M.. (2006b). Pathways of carbon assimilation and ammonia oxidation suggested by environmental genomic analyses of marine *Crenarchaeota*. PLoS Biol. 4:e95. 10.1371/journal.pbio.0040095, PMID: 16533068 PMC1403158

[ref25] Hanks J. H. Weintraub R. L. (1936). The pure culture isolation of ammonia-oxidizing bacteria. J. Bacteriol. 32, 653–670. 10.1128/JB.32.6.653-670.1936, PMID: 16559981 PMC545427

[ref26] Hatzenpichler R. (2012). Diversity, physiology, and niche differentiation of ammonia-oxidizing archaea. Appl. Environ. Microbiol. 78, 7501–7510. 10.1128/AEM.01960-12, PMID: 22923400 PMC3485721

[ref27] Heraeus W. (1886). Ueber das Verhalten der Bacterien im Brunnenwasser, sowie uber reducirende und oxydirende Eigenschaften der bacterien. Z. Hyg. 1, 193–242. 10.1007/BF02188450

[ref28] Houlton B. Z. Almaraz M. Aneja V. Austin A. T. Bai E. Cassman K. G.. (2019). A world of cobenefits: solving the global nitrogen challenge. Earth’s Future 7, 865–872. 10.1029/2019EF001222, PMID: 31501769 PMC6733275

[ref29] Kits K. D. Sedlacek C. J. Lebedeva E. V. Han P. Bulaev A. Pjevac P.. (2017). Kinetic analysis of a complete nitrifier reveals an oligotrophic lifestyle. Nature 549, 269–272. 10.1038/nature23679, PMID: 28847001 PMC5600814

[ref30] Koch R. (1882). “Die aetiologie der tuberculose. Berl. Klin. Wchnschr., xix: 221-230” in Milestones in microbiology: 1556 to 1940. ed. Brock T. D. (Washington, DC, USA: ASM Press), 109.

[ref31] Koch H. van Kessel M. A. H. J. Lücker S. (2019). Complete nitrification: insights into the ecophysiology of comammox *Nitrospira*. Appl. Microbiol. Biotechnol. 103, 177–189. 10.1007/s00253-018-9486-3, PMID: 30415428 PMC6311188

[ref32] Könneke M. Bernhard A. E. de la Torre J. R. Walker C. B. Waterbury J. B. Stahl D. A. (2005). Isolation of an autotrophic ammonia-oxidizing marine archaeon. Nature 437, 543–546. 10.1038/nature03911, PMID: 16177789

[ref33] Koops H. P. Pommerening-Röser A. (2001). Distribution and ecophysiology of the nitrifying bacteria emphasizing cultured species. FEMS Microbiol. Ecol. 37, 1–9. 10.1111/j.1574-6941.2001.tb00847.x

[ref34] Koops H. P. Purkhold U. Pommerening-Röser A. Timmermann G. Wagner M. (2006). “The lithoautotrophic ammonia-oxidizing bacteria” in The Prokaryotes. Vol. 5 eds. Dworkin M. Falkow S. Rosenberg E. Schleifer K. H. Stackebrandt E. (New York, NY: Springer), 778–811.

[ref35] Kowalchuk G. A. Stephen J. R. (2001). Ammonia-oxidizing bacteria: a model for molecular microbial ecology. Annu. Rev. Microbiol. 55, 485–529. 10.1146/annurev.micro.55.1.485, PMID: 11544365

[ref36] Müller A. (1875). “Ammoniakgehalt des Spree‐ und Wasserleitungs wassers in Berlin” in Fortsetzung der Vorarbeiten zu einer zukünftigen Wasser-Versorgung der Stadt Berlin ausgeführt in den Jahren 1868 und 1869. Berlin, Germany: Deitrich Reimer, 121–123.

[ref37] Munro J. H. M. (1886). The formation and destruction of nitrates and nitrites in artificial solution and in river and well waters. J. Chem. Soc. Trans. 49, 632–681. 10.1039/CT8864900632

[ref38] Palomo A. Fowler J. S. Gülay A. Rasmussen S. Sicheritz-Ponten T. Smets B. F. (2016). Metagenomic analysis of rapid gravity sand filter microbial communities suggests novel physiology of *Nitrospira* spp. ISME J. 10, 2569–2581. 10.1038/ismej.2016.63, PMID: 27128989 PMC5113852

[ref39] Palomo A. Pedersen A. G. Fowler S. J. Dechesne A. Sicheritz-Pontén T. Smets B. F. (2018). Comparative genomics sheds light on niche differentiation and the evolutionary history of comammox *Nitrospira*. ISME J. 12, 1779–1793. 10.1038/s41396-018-0083-3, PMID: 29515170 PMC6018701

[ref40] Pasteur L. (1862). Etudes sur les mycoderme. C. R. Acad. Sci. 54, 265–270.

[ref41] Penn M. Dworkin M. (1976). Robert Koch and two visions of microbiology. Bacteriol. Rev. 40, 276–283. 10.1128/MMBR.40.2.276-283.1976, PMID: 786252 PMC413958

[ref42] Pinto A. J. Marcus D. N. Ijaz U. Z. Bautista-de Lose Santos Q. M. Dick G. J. Raskin L. (2015). Metagenomic evidence for the presence of comammox *Nitrospira*-like bacteria in a drinking water system. mSphere 1:e00054–15. 10.1128/mSphere.00054-15, PMID: 27303675 PMC4863621

[ref43] Prosser J. I. (1989). Autotrophic nitrification in bacteria. Adv. Microb. Physiol. 30, 125–181. 10.1016/s0065-2911(08)60112-5, PMID: 2700538

[ref44] Prosser J. I. Nicol G. W. (2012). Archaeal and bacterial ammonia-oxidisers in soil: the quest for niche specialisation and differentiation. Trends Microbiol. 20, 523–531. 10.1016/j.tim.2012.08.001, PMID: 22959489

[ref45] Schleper C. Jurgens G. Jonuscheit M. (2005). Genomic studies of uncultivated archaea. Nat. Rev. Microbiol. 3, 479–488. 10.1038/nrmicro1159, PMID: 15931166

[ref46] Schleper C. Nicol G. W. (2010). “Ammonia-oxidising archaea—physiology, ecology and evolution” in Advances in microbial physiology. ed. Robert K. P. (NY, USA: Academic Press New York), 1–41. 10.1016/B978-0-12-381045-8.00001-1 21078440

[ref47] Schloesing J. J. T. Müntz A. (1877a). Sur la nitrification pas les ferments organisés. C. R. Acad. Sci. 84, 301–303.

[ref48] Schloesing J. J. T. Müntz A. (1877b). Sur la nitrification pas les ferments organisés. C. R. Acad. Sci. 85, 1018–1020.

[ref49] Stahl D. A. de la Torré J. R. (2012). Physiology and diversity of ammonia-oxidizing archaea. Annu. Rev. Microbiol. 66, 83–101. 10.1146/annurev-micro-092611-150128, PMID: 22994489

[ref50] Treusch A. H. Kletzin A. Raddatz G. Ochsenreiter T. Quaiser A. Meurer G.. (2004). Characterization of large-insert DNA libraries from soil for environmental genomic studies of archaea. Environ. Microbiol. 6, 970–980. 10.1111/j.1462-2920.2004.00663.x, PMID: 15305922

[ref51] Urakawa H. Martens-Habbena W. Stahl D. A. (2011). “Physiology and genomics of ammonia-oxidizing archaea” in Nitrification. eds. Ward B. B. Arp D. J. Klotz M. G. (Washington, DC: ASM Press), 460.

[ref52] van Kessel M. A. H. J. Speth D. R. Albertsen M. Nielsen P. H. den Camp H. J. O. Kartal B.. (2015). Complete nitrification by a single microorganism. Nature 528, 555–559. 10.1038/nature16459, PMID: 26610025 PMC4878690

[ref53] Venter J. C. Remington K. Heidelberg J. F. Halpern A. L. Rusch D. Eisen J. A.. (2004). Environmental genome shotgun sequencing of the Sargasso Sea. Science 304, 66–74. 10.1126/science.1093857, PMID: 15001713

[ref54] Waksman S. (1953). Sergei N. Winogradsky his life and work. New Brunswick, New Jersey: Rutgers University Press.

[ref55] Warington R. (1878a). Nitrification. Nature 17, 367–369. 10.1038/017367a0

[ref56] Warington R. (1878b). On nitrification. J. Chem. Soc. Trans. 33, 44–51. 10.1039/CT8783300044

[ref57] Warington R. (1879). On nitrification part II. J. Chem. Soc. Trans. 35, 429–456. 10.1039/CT8793500429

[ref58] Warington R. (1884). On nitrification part III. J. Chem. Soc. Trans. 45, 637–672. 10.1039/CT8844500637

[ref59] Warington R. (1891). On nitrification part IV. J. Chem. Soc. Trans. 59, 484–529. 10.1039/CT8915900484

[ref60] Watson S. W. Bock E. Harms H. Koops H. P. Hooper A. B. (1989). “Nitrifying bacteria” in Bergey’s manual of systematic bacteriology. Vol. 3 eds. Staley J. T. Bryant M. P. Pfennig N. Holt J. G. (Baltimore, MD: Williams and Wilkins), 1808–1834.

[ref61] Watson S. W. Valois F. W. Waterbury J. B. (1981). “The family nitrobacteraceae” in The prokaryotes. Vol. 1 eds. Starr M. P. Stolp H. Trüper H., (Berlin, Germany: Springer-Verlag), 1005–1022.

[ref62] Winogradsky S. (1887). Ueber Schwefelbacterien. Bot. Zeit. 45, 489–610.

[ref63] Winogradsky S. (1890). Sur les organisms de la nitrification. Ann. Inst. Pasteur 4, 215–231, 257–275, 760–771.

[ref64] Winogradsky S. (1891). Sur les organisms de la nitrification. Ann. Inst. Pasteur 5, 92–100, 577–616.

[ref65] Winogradsky S. (1936). The doctrine of pleomorphism in bacteriology. Soil Sci. 43, 327–340. 10.1097/00010694-193705000-00001

[ref66] Xia F. Wang J. G. Zhu T. Zou B. Rhee S. K. Quan Z. X. (2018). Ubiquity and diversity of complete ammonia oxidizers (comammox). Appl. Environ. Microbiol. 84:e01390–18. 10.1128/AEM.01390-18, PMID: 30315079 PMC6275355

[ref67] Zavarzin G. A. (2006). Winogradsky and modern microbiology. Microbiology 75, 501–511. 10.1134/S0026261706050018 17091583

[ref68] Zavarzin G. A. Legunkova R. (1959). The morphology of *Nitrobacter winogradskyi*. J. Gen. Microbiol. 21, 186–190. 10.1099/00221287-21-1-186, PMID: 13847099

